# A missing puzzle piece: Why organizational knowledge matters to public health research and practice

**DOI:** 10.3389/fpubh.2026.1784977

**Published:** 2026-07-22

**Authors:** Robert Larsson

**Affiliations:** Department of Health Sciences, Innovation, and Design, Mälardalen University, Västerås, Sweden

**Keywords:** education, knowledge, organization, practice, public health, research, theory

## Introduction

Growing up, I remember the endless search for missing puzzle and Lego pieces. Sometimes, my siblings had cruelly stolen them; other times, they had simply vanished. Many years later, while attending a doctoral course on public health, I was reminded of those missing pieces.

During the course, I asked a panel of esteemed Swedish public health professors why public health sciences rarely talk about or teach organizational matters. Discussions in the field often focus on how various factors influence health at the individual, group, community, or population level. Yet the organizational level is mostly overlooked, without explanation. It is nevertheless highly influential in people's lives through how organizations are managed, governed, and changed; the values and norms that shape their culture; and how resources are allocated.

An organization can be seen as a social and historical system where people coordinate actions and relationships within certain structures and boundaries (1). At the same time, it is constantly changing and renegotiated. Power dynamics also shape how it develops and functions. Think of a workplace or school with rules and routines, yet it is always changing as people interact, make decisions, and shape how things work. Organizations are found across all settings, and much public health work is carried out by public-sector and civil society organizations.

Thinking back on that panel discussion, I recall the panelist's darting glances and uncomfortable shifting. No one seemed eager to answer my question. After a while, one professor finally stated that occupational medicine addresses organizational matters. Although I found the answer unsatisfactory, I let it go. But it made me more curious, as I had recently read an editorial criticizing the lack of organizational theory in occupational medicine–particularly the missing link between work organization and job-level factors affecting health (2). Since then, I have continued to think about the lack of organizational knowledge in public health. With only a few exceptions, organizational theory and related knowledge are often omitted or overlooked in public health textbooks (3). Although some definitions of public health mention “organized efforts in society” (4), this phrase provides limited theoretical and practical guidance. Over time, I have seen little progress in bringing organizational knowledge–including theoretical, empirical, and practical knowledge–into the public health field, despite the profound influence organizations have on our lives.

Interestingly, organizational issues have received greater attention in related fields such as health systems research, where numerous organizational studies have been conducted in health care settings (5). While implementation science widely recognizes organizational processes, context, readiness, and leadership as critical factors in translating evidence into practice (6–8), the explicit integration of organizational theory into empirical implementation research has yet to become widespread. At the same time, a growing body of work illustrates the potential value of applying organizational theories, including institutional theory and contingency theory, to explain implementation processes and outcomes (9–11).

### Why organizational knowledge matters

The first reason is that organizational factors shape health. Drawing on Bronfenbrenner's ecological systems theory (12), factors at multiple system levels–including the organizational level–play a key role in people's health and development. At this level, organizational factors determine how work is organized and managed and act as framing conditions for the psychosocial work environment (13). Mintzberg conceptualizes organizational structure as the overall set of ways in which work is divided into distinct tasks and how these tasks are coordinated within organizations, with important implications for health (13, 14). While these organizational factors and structures can be difficult to pinpoint, most working people recognize that organizational conditions influence how they experience demands and resources in their jobs. For example, a poorly managed organizational change process can create job demands that outweigh job resources, leading to worsening working conditions, stress, and poor health.

The second reason may be less immediately relatable, but it is important. Most public health work is organized and carried out by local governments, health departments, agencies, and civil society organizations. Understanding *how* and *why* these organizations' function is crucial. Yet, as organizational scholars Tsoukas and Vladimirou note, “organizational knowledge is much talked about but little understood” (15). In public health, it is even less discussed and understood. Knowledge begins as an individual capability, but when shared in organized contexts, it becomes organizational. For example, a health practitioner working in disadvantaged neighborhoods learns through experience how to engage with hard-to-reach groups and shares these approaches with the team, which adopts them in other neighborhoods.

Put simply, organizational knowledge is the shared experience and understanding that helps organization members make better decisions based on past successes and failures (15). It interacts with individual expertise and the actions people take within organizations, making it crucial for public health. This perspective has been further developed as “knowing in practice,” which highlights the crucial role of human action in how knowledge is enacted in complex organizations. From this view, knowing is not static, embedded capability or a stable mindset among organizational members, but rather an ongoing social process that is shaped and reshaped as members interacts with others within and outside their organization (16). To succeed, public health practitioners need to understand both formal and informal aspects of organizations–how decisions are made, how change (17) and organizational politics work, and how an organization's history shapes the present. They must also navigate the organization and persuade colleagues, managers, and politicians to support their initiatives (18). From a “knowing in practice” perspective, this requires public health practitioners to activelydraw on both explicit knowledge and tacit knowledge components, including past experiences, organization-specific insights, and community contextual knowledge, while also recognizing and integrating the tacit knowledge of others in their networks (19).

This navigational skill is especially important in public-sector organizations, which some scholars describe as fundamentally different from other organizations (20). They are politically governed and part of society's political system as major political actors. Organizational understanding is thus essential for implementing effective public health policies, programs, and practices that promote health and prevent disease. Here, organizational theory can be used both retrospectively and prospectively to analyze how successful public health initiatives emerge, develop, and are implemented and evaluated within organizations (10). Institutional theory, for example, can contribute to understanding how different public health initiatives are negotiated and prioritized within organizations, and how they become increasingly similar across organizational contexts (21, 22). It can also help explain how these initiatives and their evaluation results are—or are not—granted legitimacy within the organization (23). However, beyond these theoretical perspectives, effective public health work also requires competent organizational leaders who can foster a culture and governance focused on improving and promoting equitable population health (24).

### Suggested actions

To strengthen organizational knowledge in public health, I propose the following:

- Integrate organizational knowledge into public health education. Public health curricula should include more organizational knowledge and theory, especially for students pursuing careers in public sector organizations. Encouragingly, recent public health competency frameworks (25, 26) recognize this need and show promising signs of incorporating more organizational knowledge. As educators, we should design lessons on organizational knowledge, integrate them into existing classes or create stand-alone courses, and share our teaching experiences at conferences and professional forums.
- Collaborate with organizational researchers. Public health researchers should engage and collaborate with organizational and administrative researchers when studying public health policies, programs, or practices taking place in organizational contexts such as schools, workplaces, health care settings, communities, or local governments (5, 27).
- Foster reflective public health practice. Many public health practitioners struggle with organizational change, including readiness and resistance, power struggles, and an unsupportive organizational culture. A deeper understanding of organizations helps practitioners navigate challenges and develop a more reflective approach to their work (28).

## Discussion

Most public health work takes place within organizations, and I am convinced that the public health community needs to strengthen its *collective* organizational knowledge. This is particularly important given the complex societal challenges we face, such as rising health inequities in many communities, cities, and countries. Organizational knowledge is a missing piece of the puzzle in public health education, research, and practice–one that, if added and integrated with other forms of knowledge, could contribute to more effective efforts and, ultimately, to healthier and more sustainable societies.
